# A Complementary Mechanism of Bacterial mRNA Translation Inhibition by Tetracyclines

**DOI:** 10.3389/fmicb.2021.682682

**Published:** 2021-06-28

**Authors:** Victor Barrenechea, Maryhory Vargas-Reyes, Miguel Quiliano, Pohl Milón

**Affiliations:** ^1^Faculty of Health Sciences, Centre for Research and Innovation, Universidad Peruana de Ciencias Aplicadas (UPC), Lima, Peru; ^2^Postgraduate Unit, Medicine Faculty, Universidad Nacional Mayor de San Marcos, Lima, Peru

**Keywords:** tetracycline, antibiotic, ribosome, initiation factor, tigecycline, translation initiation

## Abstract

Tetracycline has positively impacted human health as well as the farming and animal industries. Its extensive usage and versatility led to the spread of resistance mechanisms followed by the development of new variants of the antibiotic. Tetracyclines inhibit bacterial growth by impeding the binding of elongator tRNAs to the ribosome. However, a small number of reports indicated that Tetracyclines could also inhibit translation initiation, yet the molecular mechanism remained unknown. Here, we use biochemical and computational methods to study how Oxytetracycline (Otc), Demeclocycline (Dem), and Tigecycline (Tig) affect the translation initiation phase of protein synthesis. Our results show that all three Tetracyclines induce Initiation Factor IF3 to adopt a compact conformation on the 30S ribosomal subunit, similar to that induced by Initiation Factor IF1. This compaction was faster for Tig than Dem or Otc. Furthermore, all three tested tetracyclines affected IF1-bound 30S complexes. The dissociation rate constant of IF1 in early 30S complexes was 14-fold slower for Tig than Dem or Otc. Late 30S initiation complexes (30S pre-IC or IC) exhibited greater IF1 stabilization by Tig than for Dem and Otc. Tig and Otc delayed 50S joining to 30S initiation complexes (30S ICs). Remarkably, the presence of Tig considerably slowed the progression to translation elongation and retained IF1 in the resulting 70S initiation complex (70S IC). Molecular modeling of Tetracyclines bound to the 30S pre-IC and 30S IC indicated that the antibiotics binding site topography fluctuates along the initiation pathway. Mainly, 30S complexes show potential contacts between Dem or Tig with IF1, providing a structural rationale for the enhanced affinity of the antibiotics in the presence of the factor. Altogether, our data indicate that Tetracyclines inhibit translation initiation by allosterically perturbing the IF3 layout on the 30S, retaining IF1 during 70S IC formation, and slowing the transition toward translation elongation. Thus, this study describes a new complementary mechanism by which Tetracyclines may inhibit bacterial protein synthesis.

## Introduction

**FIGURE 1:**
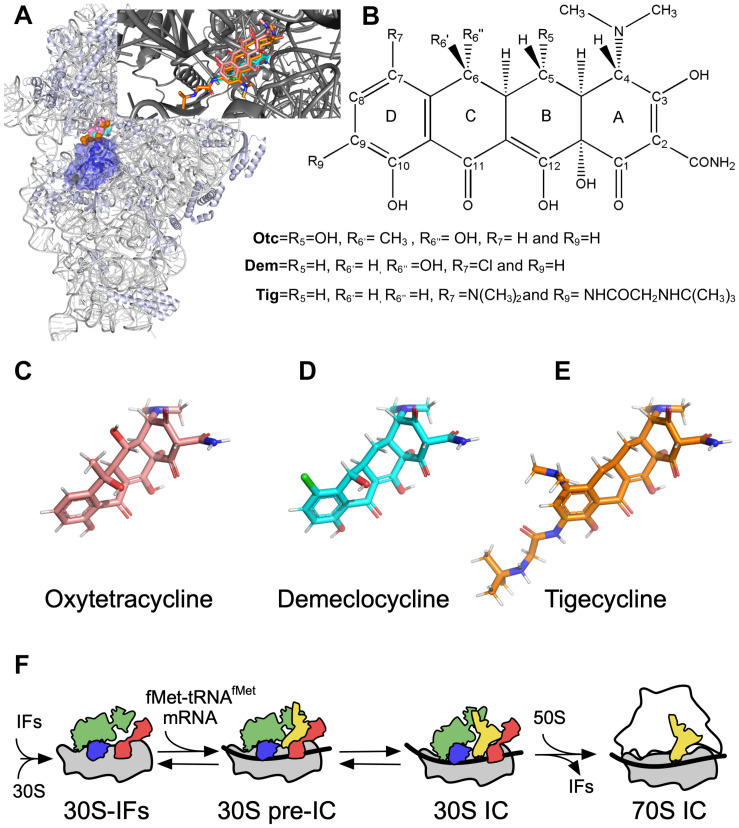
Tetracyclines and translation initiation. **(A)** Overview of 30S ribosomal subunit and binding site of Tetracyclines. IF1 is shown in blue. Inset, close up of the tetracyclines interaction with the A site. The structures are drawn from PDB 5LMV (30S complex) (Hussain et al., 2016), and PDB 4YHH was used to align all Tetracyclines in the inset. **(B)** Tetracycline core chemical structure. **(C)** Oxytetracycline (Otc, pink). **(D)** Demeclocycline (Dem, sky blue). **(E)** Tigecycline (Tig, orange). **(F)** Scheme of the main steps of translation initiation in bacteria. IF3 (red), IF2 (green), and IF1 (blue) bind the 30S to form the 30S–IFs complex. Binding of the mRNA (black ribbon) and fMet-tRNA^fMet^ (yellow) build the 30S pre-IC, which, upon decoding the start codon, rearranges to the 30S IC. The large 50S subunit can bind the 30S IC, triggering the dissociation of IFs and entering the elongation phase of protein synthesis.

Tetracycline, discovered in the ’40s, was initially extracted from *Streptomyces aureofaciens* and then produced by synthetic processes (Chopra and Roberts, 2001). In humans, Tetracyclines are mainly used to treat acne and other skin complications caused by bacteria (Kligman, 2014). Yet, the WHO recommends it as an alternative application for the treatment of gastritis and preventively against *gonococcal* conjunctivitis in neonates. Tetracyclines were commonly used to treat respiratory diseases, such as pneumonia (Eliopoulos and Roberts, 2003). Tetracyclines are also an important pillar in the veterinary industry, acting as a growth promoter for livestock and aquaculture (Chattopadhyay, 2014; Hao et al., 2014). Beekeepers also use them to treat diseases in honeycombs (Marshall and Levy, 2011). The remarkable effectiveness of this family of antibiotics is due to their broad bactericidal spectrum, including gram-positive and negative bacteria and parasites (Chopra and Roberts, 2001). It should be noted that Tetracyclines, today, are low-cost antibiotics with increased availability and use in diverse industries (Michalova et al., 2012). Tetracyclines are bactericidal drugs that inhibit protein synthesis by binding the A site of the small 30S subunit (Connamacher and Mandel, 1965; Suzuka et al., 1966; Blanchard et al., 2004; Giuliodori et al., 2019; Figure 1A).

The tetracyclines discovered during the Golden Age of antibiotics are known as the first-generation and comprised Tetracycline (Tet) and Oxytetracycline (Otc). Then, Doxycycline and minocycline were developed to respond to the rising of antibiotic resistance (Jarolmen et al., 1970; Cunha et al., 1982; Wright, 2011). Third-generation tetracyclines, the Glycylcyclines, contain an *N*-alkyl-glycylamido group at C9, which allow efficient interaction with the ribosome and a potent activity against multidrug-resistant pathogens (Petersen et al., 1999; Livermore, 2005; Slover et al., 2007). Tigecycline (Tig) causes a limited effect on the conformation of the repressor protein TetR, thus blocking the efflux pump (Hirata et al., 2004), and the C9-moiety sterically interrupts the RPP Tet(M) and the displacement of the drug from its binding site (Jenner et al., 2013).

Tetracyclines bind to the 30S subunit, specifically at the A site (Figure 1A). The structural core of tetracyclines comprises four aromatic rings called DCBA naphthacene that clashes sterically with tRNAs, mainly with the C and D ring (Figure 1B; Nguyen et al., 2014). The Tetracycline derivatives vary from the structural core essentially by chemical modifications in Otc (C5 and C6), Dem (C6 and C7), and Tig (C7 and C9) (Figures 1B–F). The molecular mechanism of Tetracycline-mediated translation elongation inhibition was proposed by early biochemical studies and later by more sophisticated structural and single-molecule reports (Suzuka et al., 1966; Blanchard et al., 2004; Jenner et al., 2013). Crystallization of the 30S and tetracycline uncovered the detailed atomic interactions in the A site and other secondary interactions (Pioletti et al., 2001; Jenner et al., 2013). Single-molecule assays demonstrated that tetracycline blocked the A site even if the aminoacyl-tRNA was delivered by EF-Tu (Blanchard et al., 2004). Thus, the consensus mechanism of tetracycline-mediated inhibition of bacterial translation indicates that the antibiotic prevents the binding of aminoacyl-tRNAs to the A site, inhibiting the elongation phase.

In addition to the elongation phase, the 30S A site orchestrates essential reactions in other phases of the bacterial protein synthesis. Particularly, crystallographic and Cryo-EM structures of 30S complexes located IF1 in the A site, near the interaction sites of tetracyclines in the small 30S subunit (Carter et al., 2001; Pioletti et al., 2001; Simonetti et al., 2008; Julián et al., 2011; Jenner et al., 2013; Hussain et al., 2016; López-Alonso et al., 2017). Co-crystallization and biochemical characterization of Sarecycline, a novel derivative of tetracycline, with 70S ribosomes, mRNA, and tRNA^Met^ at the P site suggested that the mechanism of inhibition was occurring before the elongation phase of protein synthesis (Batool et al., 2020). Additionally, *in vivo* experiments of ribosome profiling that used tetracycline to stop translation showed increased read densities around mRNA start sites (Nakahigashi et al., 2016). Tetracycline also inhibited IF3 functions *in vitro*, resulting in the destabilization of translation initiation complexes (Risuleo et al., 1976). Thus, Tetracyclines bind to the A site of the 30S subunit and inhibit translation elongation, yet observations are suggesting that tetracyclines can also act at earlier steps of protein synthesis. The molecular mechanism of translation initiation inhibition by Tetracyclines remained largely unexplored. This study uses pre-steady state kinetics techniques and molecular modeling to study how tetracyclines affect different steps of translation initiation. The data gathered unveil a novel mechanism of action of tetracyclines during early steps of protein synthesis that can be further exploited for the rational design of next-generation compounds.

## Results

### Tetracyclines Promote a Compact Conformation of the 30-Bound IF3

**FIGURE 2:**
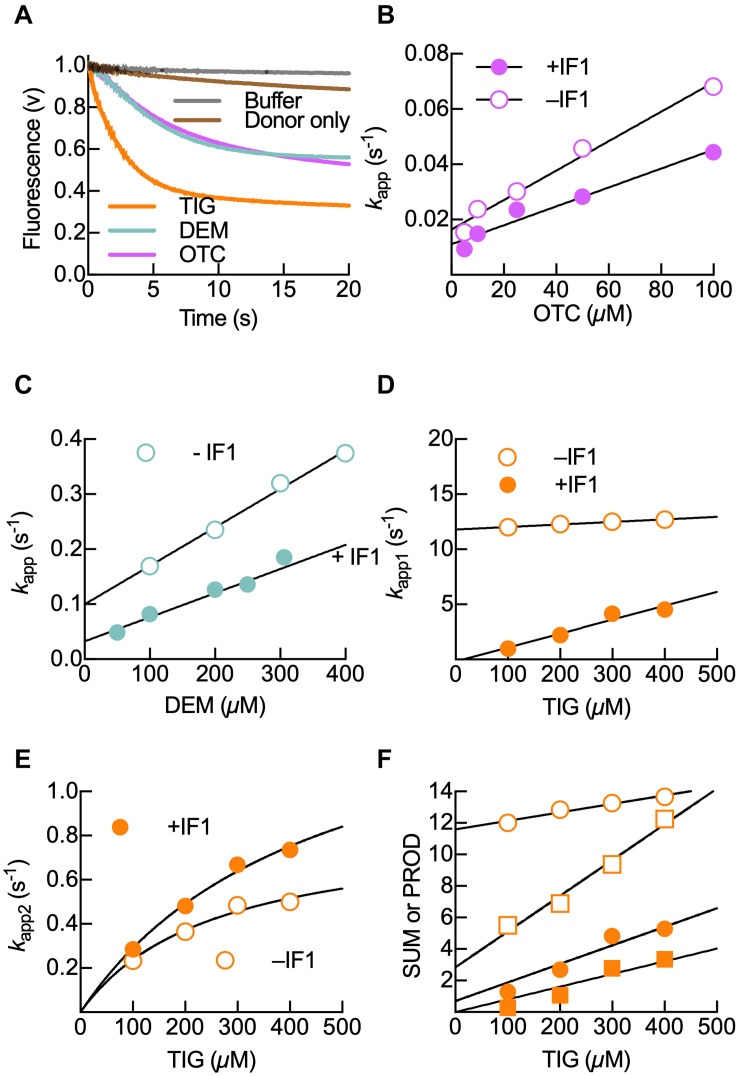
Kinetics of 30S-bound IF3_DL_ compaction upon binding of Otc, Dem, and Tig. **(A)** Time courses of IF3_DL_ fluorescence change during binding of 100 μM Tig (orange), Dem (aquamarine), or Otc (Pink) to 0.05 μM 30S–IF3_DL_ complexes. No acceptor control is shown in brown, while the gray trace represents the dilution control upon mixing 0.05 μM 30S IF3_DL_ complexes with buffer. Shown time courses result from six to eight averaged replicates. Apparent rate (*k*_app_) dependence on Otc **(B)**, Dem **(C)**, and Tig **(D,E)** in the absence (open circles) or the presence (full circles) of 0.15 μM IF1. Continuous lines in panels **(B–D)** show linear regressions of the bimolecular encounter of tetracyclines with the corresponding complex. The continuous lines in panel **(D)** show a hyperbolic fitting for the accommodation step observed for Tig binding. **(F)** Analytical solution for the mechanism IF3_DL_ conformational change upon Tig interaction with the 30S subunit using a two-step model. Plots of the sum (circles) and product (squares) of *k*_app1_ and *k*_app2_ linearly depended on Tig concentration, allowing to calculate all four microscopic constants (*k*_1_, *k*_–1_, *k*_2_, and *k*_–2_) (see section “Materials and Methods”). Error bars in panels **(B–F)** indicate standard errors of the fit.

IF3 prevents the association of the 30S subunit to the 50S subunit, thus providing free 30S ribosomal subunits to initiate translation (Wishnia et al., 1975). Direct measurements using rapid kinetics and föster resonance energy transfer (FRET) showed that IF3 binds before IF2 and IF1 (Milon et al., 2012). IF3 consists of two domains, IF3N and IF3C, joined by a highly flexible lysine-rich connector, and binds independently to the 30S subunit (Biou et al., 1995; Sette et al., 1999; Fabbretti et al., 2007; Julián et al., 2011; Hussain et al., 2016). Tetracyclines bind near the reported site for IF1, at the A site. Thus, the binding of tetracyclines to the 30S subunit could influence the interaction between IF1 and IF3 with the ribosomal subunit. In order to inquire into this postulate, we used a fluorescent double-labeled IF3 (IF3_DL_). IF3_DL_ contains an Alexa-488 fluorophore (donor) in IF3N and Atto-540Q (silent acceptor) in IF3C, allowing to monitor inter-domain distance changes as measured by FRET (Chulluncuy et al., 2016; Nakamoto et al., 2019). The 30S subunit pre-bound to IF3_DL_ was mixed in a Stopped-flow apparatus with different concentrations of Oxytetracycline (Otc), Demeclocycline (Dem), or Tigecycline, and FRET was monitored in time by measuring donor fluorescence (Figure 2A and Supplementary Figure 1).

The binding of all tetracyclines to 30S–IF3_DL_ resulted in reducing IF38 interdomain distances, albeit with differences in the magnitudes and kinetics (Figure 2A and Supplementary Figure 1). Otc and Dem trigger an interdomain distance reduction of IF3 that is about 50% of that caused by Tig (Figure 2A). Also, antibiotic titrations show that the IF3 distance reduction depends on the antibiotic concentration in a tetracycline-dependent manner (Supplementary Figure 1). Binding of Dem or Otc (Figures 2B,C) to the IF3_DL_–30S complex showed that the IF3 conformational changes were slower than that caused by Tig (Figures 2D,E and Table 1). Altogether, binding of tetracycline and its derivatives to the early 30S–IF3 complex promotes the factor to acquire a conformation where IF3 domains are closer than in the absence of the antibiotics (Figure 2 and Supplementary Figures 1, 3A). A similar compaction of the 30S-bound IF3 was observed when IF1 was binding (Elvekrog and Gonzalez, 2013; Chulluncuy et al., 2016). This change in interdomain distance is likely to arise by promoting the IF3C displacement toward the P site (Hussain et al., 2016; Nakamoto et al., 2019).

**TABLE 1:** Summary of rate constants of IF3 conformational changes during binding of Tetracyclines to 30S subunit.

	30S–IF3_DL_ complex	*k*_1_ (μM^–1^ s^–1^) × 10^–3^	*k*_–1_ (s^–1^) × 10^–3^	*k*_2_ (s^–1^)	*k*_–2_ (s^–1^)	*K*_D_ (μM)*
Otc^†^	−IF1	0.53 ± 0.04	15 ± 2	–	–	31 ± 2
	+IF1	0.34 ± 0.04	11 ± 2	–	–	33 ± 2
Dem^†^	−IF1	0.7 ± 0.02	100 ± 7	–	–	143 ± 10
	+IF1	0.44 ± 0.05	32 ± 9	–	–	74 ± 9
Tig^§^	−IF1	5 ± 1	7,318 ± 329	3.8 ± 0.4	0.38 ± 0.1	124 ± 10
	+IF1	12 ± 2	16 ± 4	0.68 ± 0.1	≈0	≪1

^†^Derived from a single-step interaction model between the tetracycline and the complex (Eq. 1). ^§^ Derived from a two-step interaction model between Tig and the 30S complexes (Eq. 2). *The *K*_D_ for Otc and Dem = *k*_–1_/*k*_1_. For Tig, *K_D_* = (*k*_–1_ × *k*_–2_ × *k*_1_^–1^)/(*k*_–2_ + *k*_2_).

### Tetracyclines and IF1 Cooperatively Induce IF3 Compaction

Aside from monitoring the effect of antibiotics on the 30S–IF3_DL_ complex, a similar titration test was performed in the presence of IF1 (Figure 2 and Supplementary Figure 1). As observed for the complex lacking IF1, donor fluorescence decreased in time upon tetracycline binding (Supplementary Figure 1). However, the amplitude changes caused by the antibiotics were smaller if compared to the absence of IF1 (Supplementary Figure 2). This may reflect that IF3 domains were already nearby due to the binding of IF1. The pre-binding of Otc with the 30S subunit did not perturb IF1 binding as measured from FRET between IF1_540Q_ and IF3_488_ (Supplementary Figure 3B). Similarly, Otc did not disturb the arrival of IF3_540Q_ to 30S subunits preincubated with an mRNA labeled with Alx488 (Supplementary Figure 3C). Altogether, tetracyclines do not affect IF1, nor IF3 binding, yet the IF3 layout on small ribosomal subunit seems to be affected. Thus, tetracyclines and IF1 appear to cooperatively promote an IF3 layout where its domains are proximal to each other, increasing the compaction of the factor.

In order to inquire on the molecular mechanism ruling IF3 accommodation, we analyzed the kinetics of the conformational changes as a function of antibiotic concentration and the presence or absence of IF1 (Figure 2). From titrations, the signals corresponding to the approaching domains were fit by non-linear regression using a single exponential function for Otc and Dem (Figures 2B,C). Tig time-courses appeared biphasic, and two exponential terms were used (Figures 2D–F). The analysis allowed us to obtain apparent rate constants (*k*_app_) and FRET amplitude terms for each experimental condition. We found that the *k*_app1_ values increased linearly with the concentration of tetracyclines, indicating that IF3_DL_ monitors the bimolecular interaction between the 30S subunit and the antibiotics. From de linear dependence, the forward rate constant *k*_1_ and the reverse rate constant *k*_–1_ were obtained from the slope and *y*-axis intercept of Otc and Dem titrations, respectively (Figures 2B,C and Table 1). On the other hand, Tig binding was best described by two sequential reactions, the initial binding followed by a further rearrangement of IF3_DL_ (Figures 2A,D,E). To solve the elemental constants describing the mechanism of Tig-dependent IF3_DL_ rearrangement on the 30S subunit, the sum and product of *k*_app1_ and *k*_app2_ were plotted against Tig concentrations (Figures 2E,F). The linear dependencies of the sum of apparent rates allow calculating a slope and an intercept, corresponding to the *k*_1_ rate and the sum of *k*_–1_, *k*_2_, and *k*_–2_ rates, respectively. The product of the apparent rates also depended linearly with ligand concentration, allowing the calculation of the *k*_1_ rate multiplied by the sum of *k*_2_ and *k*_–2_ (slope). The intercept corresponds to the product of *k*_–1_ by *k*_–2_. Using the sum and prod of the apparent rates allowed us to estimate all four microscopic constants (*k*_1_, *k*_–1_, *k*_2_, *k*_–2_) that describe the reaction for complexes lacking IF1 (Table 1). However, in the presence of IF1, reverse reactions appeared greatly affected, and *k*_–2_ approximated to zero, preventing us to precisely calculate it (Table 1).

The forward rate binding constant *k*_1_ for Otc and Dem in the absence or presence of IF1 fluctuated between 0.3 and 0.7 × 10^–3^ μM^–1^ s^–1^, suggesting that none of the tetracycline derivatives nor IF1 influence the initial bimolecular interaction. However, the dissociation rate constant *k*_–1_ was higher for Dem than for Otc in the absence of IF1, and it was reduced in the presence of the factor for Dem only (Figures 2B,C and Table 1). The *k*_1_ and *k*_–1_ values obtained from Tig titrations were the highest among the three antibiotics, indicating that Tig binds 20- to 40-fold faster than the other tetracyclines and dissociates more easily from the initial bimolecular interaction (*k*_–1_, Table 1 and Figure 2D). However, if IF1 was present, the dissociation rate *k*_–1_ was reduced by over 400-fold. Similarly, the reverse accommodation rate *k*_–2_ was significantly reduced by the presence of IF1 (over 100-fold, Table 1). Interestingly, the forward *k*_2_ was slightly affected by IF1, a fivefold speed reduction (Table 1).

**FIGURE 3:**
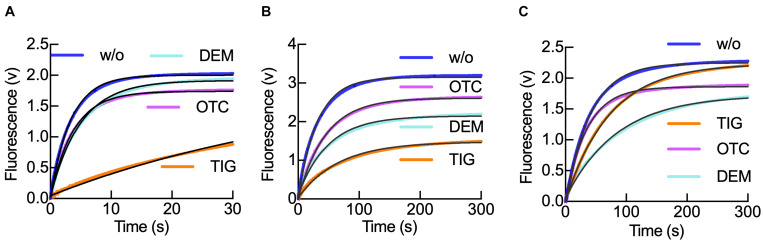
Tetracyclines modulate IF1 retention in the 30S subunit during translation initiation progression. **(A)** Time courses of IF1_540Q_ dissociation from 0.05 μM 30S–IF3_488_–IF1_540Q_ complexes upon mixing with 0.5 μM of unlabeled IF1 in the absence (blue) or in the presence of either Otc (pink), Dem (aquamarine), or Tig (orange). **(B)** As panel **(A)** for 0.05 μM 30S pre-initiation complexes (30S pre-IC) using the same FRET pair and in the presence of 0.15 μM IF2, 0.3 μM mRNA, and 0.2 mM GTP. **(C)** As panel **(B)** for the 30S IC formed by the addition of 0.15 μM fMet-tRNA^fMet^ followed by 30 min incubation at 37°C. Shown time courses result from six to eight averaged replicates. Continuous black lines represent the non-linear fitting with a single exponential term (Eq. 1) for a single-step model of IF1 interaction with the 30S subunit (Milon et al., 2012). The resultant dissociation rate constants *k*_–1_ are shown in Table 2.

Altogether, our results show that the presence of IF1 does not primarily influence the binding rate constant *k*_1_ of the three tetracyclines. On the other hand, the dissociation rate constant *k*_–1_ varies depending on the antibiotic used and IF1. While IF1 did not vary the *k*_–1_ for Otc (Figure 3B), *k*_–1_ constants decreased around 70% in the complex with Dem, and in the presence of Tig, both reverse reactions, *k*_–1_ and *k*_–2_, were drastically reduced (Table 1).

The above analysis allowed us to compare the stability of these complexes and to calculate the equilibrium constants and how the tetracyclines perturb the forward and backward conformational changes of IF3_DL_ (Table 1). We observed that the equilibrium constant for the Dem-bound 30S complex without IF1 was nearly twofold higher than when the factor was bound, indicating that IF1 and Dem cooperatively enhanced each other. To a larger extent, Tig reduced the equilibrium constant if IF1 was added to the complex by at least 100-fold. Altogether, our results indicate that IF1 increases the affinity of Dem and Tig on the 30S subunit by inferring primarily on the dissociation rate constant of the tetracyclines. Interestingly, Otc appears kinetically unaffected by IF1 but retains its capability to promote IF3 closure on the 30S subunit.

### Tetracyclines and IF1 Along the Pathway of Initiation

Translation initiation in prokaryotes comprises three main steps. The first step involves the 30S pre-initiation complex (30S pre-IC), composed of the 30S subunit, the three initiation factors, mRNA, and fMet-tRNA^fMet^, yet the codon–anticodon interaction is still missing (Gualerzi et al., 2010; Gualerzi and Pon, 2015). After conformational changes that allow codon–anticodon base-pairing, a locked 30S initiation complex (30S IC) is built. Finally, the 50S subunit can join the 30S IC to form a 70S initiation complex (70S IC) (Figure 1F). As noted in the previous results, both IF1 and tetracyclines cause similar responses in the conformation of IF3_DL_. Remarkably, IF1 increased the affinity of Tig for the 30S–IF3 complex by more than 100-fold (Table 1). To further explore the cross-cooperation between tetracyclines and IF1 on intermediate 30S complexes, we directly measured the dissociation rate constant of the factor by chase experiments in a stopped-flow apparatus. Thus, 30S–IF3^488^–IF1_540Q_–Tetracycline complexes were mixed with a 10-fold excess of unlabeled IF1 (Figure 3), and donor fluorescence was measured in time. A decrease in fluorescence indicates binding, whereas an increase indicates the dissociation of IF1_540Q_ (Supplementary Figure 4).

The time course of IF1 dissociation was monophasic, in agreement with measuring the dissociation rate constant *k*_–1_ of the factor as previously described (Milon et al., 2012; Figure 3). The dissociation rate constants of IF1 from the 30S–IF1–IF3 complexes were 0.26 s^–1^ (Otc), 0.2 s^–1^ (Dem), 0.02 (Tig), and 0.3 s^–1^ in absence of tetracyclines (Table 2). Thus, Tig decreases IF1 *k*_–1_ by 14-fold, while Dem and Otc only slightly affected it (Figure 3A and Table 2). Comparison of amplitudes of FRET changes showed the impact of Tig on IF1 dissociation (Figure 3A). These results suggest that the third-generation tetracycline Tig retains IF1 and could compromise IF1-dependent reactions in the early 30S–IF1–IF3 complex.

**TABLE 2:** IF1 dissociation rate constants *k*_–1_ in the presence of Tetracyclines.

	*k*_–1_ (s^–1^)*			
Complex	No Tet control	Otc	Dem	Tig
30S–IF1–IF3	0.3 ± 0.01	0.26 ± 0.01	0.2 ± 0.01	0.021 ± 0.001
30S pre-IC	0.03 ± 0.001	0.02 ± 0.001	0.02 ± 0.001	0.015 ± 0.001
30S IC	0.02 ± 0.001	0.03 ± 0.002	0.012 ± 0.001	0.015 ± 0.001

*The dissociation rate constant *k*_–1_ was obtained by non-linear fitting with a single exponential term (Eq. 1) of IF1 chase experiments of the indicated complexes, as described in Milon et al. (2012).

To explore if a similar case applies to later complexes along the initiation pathway, we monitored IF1 dissociation in the primary two 30S intermediate complexes: 30S pre-IC and 30S IC (Figures 3B,C). To facilitate the formation of the 30S pre-IC, we mixed 30S subunits, the three initiation factors, and mRNA containing an AUG as start codon. For 30S IC formation, we added fMet-tRNA^fMet^ to the mixture. The formation of a 30S pre-IC significantly stabilizes IF1, and the effects of tetracyclines appear hindered in kinetic terms (Table 2). Yet, evident amplitude differences are observed, suggesting a conformational partitioning of 30S pre-ICs, likely indicating the presence of complexes that have IF1 locked (Figure 3B). In the 30S pre-IC, the amplitudes obtained were diverse for each tetracycline used. An amplitude reduction of 16 and 33% was obtained due to Otc and Dem’s presence, respectively. Tig instead caused an amplitude decrease of nearly 50% (Figure 3B). The *k*_–1_ values obtained for IF1 dissociation from the 30S pre-IC were 0.02 s^–1^ in the presence of Otc or Dem and 0.015 s^–1^ if Tig was used. Both Otc and Dem did not cause a significant decrease of the dissociation rate constant of IF1; however, Tig was nearly twofold slower compared to the absence of tetracyclines (0.03 s^–1^) (Figure 3B). Thus, the cooperative stabilization of IF1 induced by the 30S pre-IC ligands appears to hinder the effect of Tetracyclines observed in the early 30S–IF1–IF3 complex (Table 2).

Concerning IF1 dissociation from 30S ICs, Tig produced a similar amplitude to the complex in the absence of Tetracyclines; however, Dem yielded the largest reduction of amplitude, around 30% (Figure 3C). Considering the kinetics, only Dem and Tig showed a decrease in the dissociation rate constant (from 0.02 to 0.012 s^–1^ and 0.015 s^–1^, respectively) (Table 2). This complex features a different behavior of IF1 in the presence of Dem; IF1 dissociation rates appear more delayed if compared to Tig. Interestingly, the formation of the 30S IC appears to overcome Otc-dependent effects in IF1, showing a slightly faster *k*_–1_ (0.3 s^–1^) (Figure 3C and Table 2).

### Tigecycline Delays 70S IC Formation and Impairs IF1 Dissociation

**FIGURE 4:**
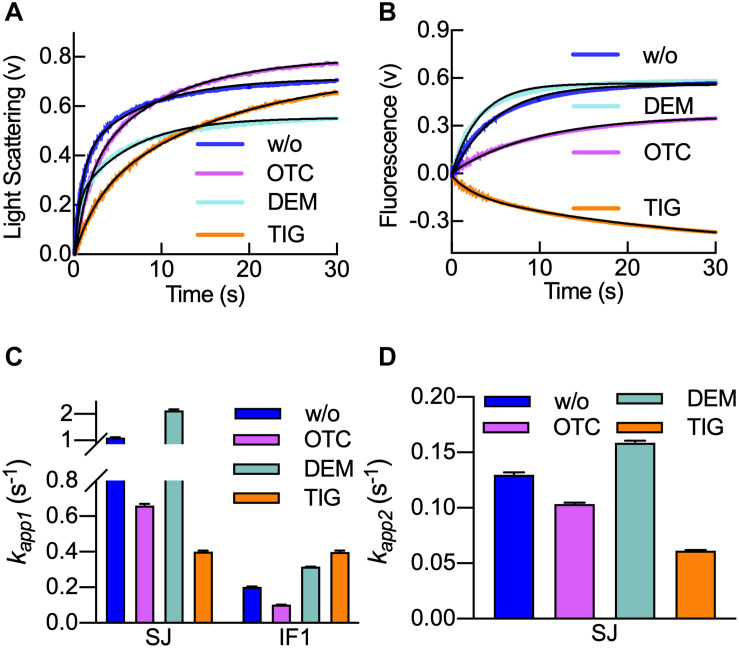
Tigecycline delays 70S IC formation and hampers IF1 dissociation. **(A)** Light scattering time courses of 0.1 μM 30S IC binding to 0.15 μM 50S in the absence of tetracyclines (blue) or the presence of Otc (pink), Dem (aquamarine), or Tig (orange). **(B)** Time courses of IF1_540Q_ dissociation during 70S IC formation as measured by FRET from fMet-tRNA^fMet^ (Flu). Colors and concentrations are as in panel **(A)**. Bar-graph comparing the apparent rates *k*_app1_ **(C)** of subunit joining and IF1 dissociation and *k*_app2_ **(D)** for subunit joining. Colors in panels **(C,D)** are as in panel **(A)**. Shown time courses result from six to eight averaged replicates. Continuous black lines in panel **(A)** represent the non-linear fitting with a double exponential term equation (Eq. 2), while in panel **(B)**, a single-step model applied for IF1_540Q_ dissociation was used (Eq. 1). Error bars in panels **(C,D)** indicate standard errors of the fit.

The rate of 50S subunit association to the 30S IC in the presence of tetracyclines is not known. Thus, we measured the kinetics of subunits joining by light scattering in the stopped-flow apparatus (Figure 4A). Large 50S subunits were mixed with 30S ICs in the presence or absence of tetracyclines, and light scattering was measured in time (Milon et al., 2007; Figure 4A). The same reaction was used to study the dissociation of IF1 from the resulting 70S ICs. For this porpoise, we used fMet-tRNA^fMet^ labeled with fluorescein (fMet-tRNA^fMet^ Flu) as a fluorescence donor and IF1_540Q_ as a non-emitting acceptor. In this case, we can monitor IF1 dissociation by an increase of fMet-tRNA^fMet^ Flu fluorescence in time (Figure 4B).

As described previously, the increase of light scattering signals was biphasic (Milon et al., 2008). The resulting *k*_app1_ and *k*_app2_ were used to compare all three tetracyclines (Figures 4C,D). As seen in Figure 4A, Tig and Otc slowed 50S subunit association with the 30S IC, while Dem appeared to enhance the reaction. A comparison of the apparent rates indicates that Otc and Tig reduce the initial 50S interaction (as seen by the *k*_app1_) by two and threefold, respectively. On the other hand, Dem appeared to increase the *k*_app1_ by twofold (Figure 4C). A similar effect is found for the *k*_app2_, with Otc and Tig reducing the rates, while Dem slightly increased it (Figure 4D). Altogether, the analysis shows that the impact of tetracyclines was greater in the first step of 70S formation than in the second one, indicating that the 30S ICs are less fit for accepting the 50S. Otc and Tig appear to compromise an efficient subunit joining, thus delaying the pathway toward translation elongation.

When IF1 dissociation was monitored upon 50S joining with the 30S IC, exponential fluorescence increasing traces were observed in the presence of Otc and Dem, thus indicating the ejection of IF1 from 30S IC due to the arrival of 50S (Figure 4B). In the presence of Tig, a fluorescence decrease was observed, suggesting that the distance between IF1 and tRNA initiator would be reduced rather than increased due to dissociation (Figure 4B). These results indicate that Tig prevents IF1 dissociation from the 30S IC, even when the 50S subunit is joining to the 30S IC. Fitting of the fluorescence time-course showed an apparent rate similar to previous reports of fMet-tRNA^fMet^ accommodation in a similar experimental setup (Goyal et al., 2015; Vinogradova et al., 2020; Figure 4C, orange bars). Thus, Tig seems to prevent IF1 dissociation from the 70S IC.

### Tig and Dem Show Enhanced Contacts With the 30S and IF1

**FIGURE 5:**
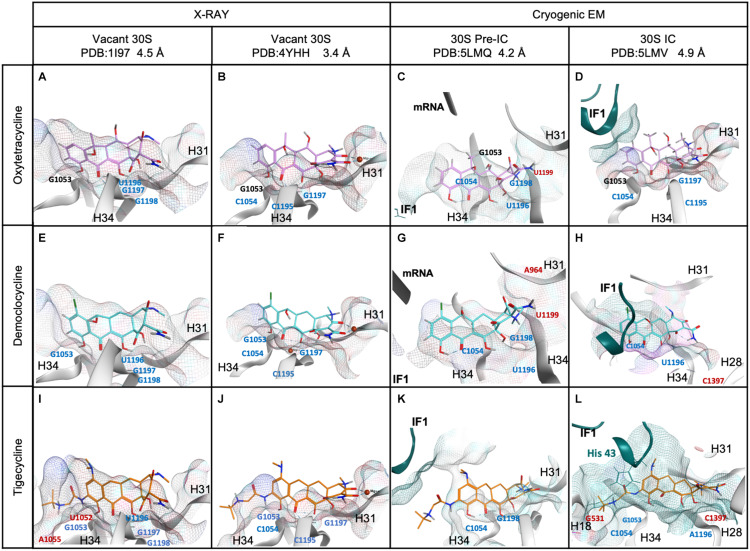
Comparison of Tetracyclines in 30S complexes obtained by X-ray diffraction, cryogenic electron microscopy, and molecular modeling approach. **(A–D)** Oxytetracycline, **(E–H)** Demeclocycline, and **(I–L)** Tigecycline. Labeled nucleotides in 16S (gray ribbon), mRNA (black ribbon), red labeled represent nucleotides that appeared once, blue show their appearance in two or three models, and black indicates that the contact was present in all 30S models of the indicated tetracycline. IF1 is shown in dark aquamarine ribbons, while interacting residues are shown in sticks. The structural models were generated from PDBs 1I97 and 4YHH for vacant 30S subunits, 5LMQ, and 5LMV for the 30S pre-IC and IC, respectively.

Our kinetic analysis allowed us to propose that tetracyclines cooperatively with IF1 promote a compact IF3 layout on the 30S, reduce IF1 dissociation, and delay 70S IC formation. Nevertheless, the extent and tetracycline dependence varied as a function of the 30S intermediate complex. i.e., Tig maximized IF1 kinetic stability on early 30S complexes, but the effect was partially lost in the 30S IC (Figure 3). On the contrary, Dem showed a maximal effect on IF1 for late complexes only (Figure 3C) and was lost during 70S IC formation (Figure 4). To investigate the molecular interactions in the 30S complexes that may explain our observation, we modeled the molecular network of interactions between all three tetracyclines in two intermediate complexes, the 30S pre-IC (PDB ID 5LMQ) and 30S IC (PDB ID 5LMV) (Hussain et al., 2016), and existing crystal structures of the vacant 30S with Tetracycline or Tigecycline (PDBs: 1I97 and 4YHH) (Pioletti et al., 2001; Schedlbauer et al., 2015; Figure 5). Our structural modeling of the 30S subunit, pre-initiation, and initiation complex showed that Otc, Dem, and Tig interacted mainly with h31 (963–966) and h34 (1052–1055, 1195–1199), in agreement with previous investigations, providing a solid analytical setup (Figure 5 and Supplementary Figure 5). The interactions presented by Tig, Otc, and Dem were highly conserved in the vacant 30S (Figure 5). Hydrogen bonds with nucleotides G1053, C1195, and G1197 and the arene interaction with C1054 should be highlighted as typical for all tetracyclines (Figure 5 and Supplementary Figure 5).

Besides the common interactions, additional contacts were either tetracycline- or 30S complex-dependent (Figure 5). Otc interacted with residues located at helixes h31 and h34 in vacant 30S and 30S IC (Figures 5A–D). Spatial arrangements of the 30S pre-IC involved mainly a change in the h31 position (Figure 5C). The residue U1196 forms two hydrogen bonds to the OH at C6 and the O of the carbonyl group at C11 (Figure 5C and Supplementary Figure 5C). Also, the residue G1198 participates in three hydrogen bonds to the H and NH of the amide group at C2 and the NH of the tertiary amine at C4. At C4 of Otc, U1199 forms an ionic interaction with the nitrogen positively charged in this last position. Otc in the vacant 30S and 30S IC shared identical interactions (G1053, C1054, C1195, and G1997) (Figures 5A,B,D), but the 30S pre-IC presented differences (G1053, C1054, U1996, G1198, and U1199) (Figure 5C). The differential contacts of Otc with the 30S pre-IC appear not to influence IF1 dissociation (Figure 3), yet they may be important during 70S IC formation, slightly delaying the reaction, and IF1 dissociation (Figure 4).

In addition to the core contacts of tetracyclines, Dem established ionic interactions between the NH group at C4 and C1054 for the 30S pre-IC (Figures 5E–H). From the structural analyses and based on the chemical structure of Dem in the 30S IC model, the residue C1054 formed two hydrogen bonds with the OH group at C10 and O of the carbonyl group at C11 (Figure 5H). The 30S IC reported the formation of a pocket between the helices h31, h34, and IF1, while h28 appeared near Dem (Figure 5H). Additionally, two exclusive molecular interactions were observed for Dem in the 30S IC (Figures 5G,H and Supplementary Figures 5G,H). The first involved an ionic interaction between the residue A964 located in h31 and NH at C4 of Dem for 30S pre-IC. The second involved a hydrogen bond between the residue A1397 located in h28 and O of the carbonyl group at C12 for 30S IC. Therefore, Dem showed important and characteristic molecular interactions with helices h31 and h28 in the 30S IC, likely explaining the retention of IF1.

Our modeling at the 30S pre-IC showed that Otc and Dem share nucleotide interactions. For both antibiotics, residue U1196 formed two hydrogen bonds at C6 and C11 of the tetracycline scaffold, while U1199 forms an ionic interaction with positively charged nitrogen (Figures 5C,G). These observations could explain the stabilization of IF1 by these antibiotics, yet through allosteric modulation of the A site. However, Tig does not present the interactions mentioned above despite having a marked effect on IF1 retention in early 30S complexes compared to Dem and Otc (Figures 3A,B, 5I–L). Tig showed a vicinity to the mRNA at position U40 (3.7 Å), located near IF3C when positioned in the P site (30S pre-IC, Figure 5K). Tig also presents exclusive interactions with helix 18 (U531 and C532) in the 30S IC (Figure 5L). Dem and Tig share interactions with helix 34 (C1054, U1196) and, for the first time, reported with helix 28 (C1397) (Figures 5H,L). These improved contacts may result in greater IF1 stabilization in 30S IC complexes, as evidenced by kinetic results (Figure 3C). However, for the formation of 70S ICs, Tig shows a more significant impact than Dem, abolishing IF1 dissociation (Figure 4B). The interaction of IF1 in the 30S IC with Tig reveals potential contacts with six amino acids of IF1 (Lys39, Met42, His43, Tyr44, Ile45, and Arg70); Dem presents 3, and none for Otc (Figure 5). Thus, the additional tert-butyl amide glycol side chain in Tig enhances IF1 retention in the 30S and 70S ICs. Specifically, the chemical modification in Tig C9 triggered an arene interaction of IF1 with histidine 43 in addition to the electrostatic interaction with h18 at U531 (Figure 5L). Therefore, the effect of Tig in intermediate complexes of the 30S IC could be explained by its exclusive contacts with IF1.

## Discussion

Tetracyclines are known inhibitors of the translation elongation phase of protein synthesis. In the early ’60s, tetracycline was shown to prevent polypeptide synthesis in cell-free extracts (Franklin, 1963; Laskin and Chan, 1964) by binding the 30S ribosomal subunit and preventing tRNA binding with a same-site competition mechanism (Connamacher and Mandel, 1965; Suzuka et al., 1966). More recent and more sophisticated experimental approaches showed that tetracycline prevents aminoacyl-tRNA binding even if delivered by EF-Tu (Blanchard et al., 2004). Crystallographic studies showed at atomic resolution that tetracycline interacts with the A site and other secondary positions in the 30S and the 50S (Pioletti et al., 2001; Jenner et al., 2013). Yet, there is also evidence indicating that the antibiotics could interfere with earlier phases. Tetracycline inhibited IF3, promoted the disassembly of 30S–mRNA–fMet–tRNA ternary complexes (Risuleo et al., 1976), and prevented ribosomal initiation complexes (Mukundan et al., 1968). More recently, using tetracycline to stall 70S ribosomes for *in vivo* Ribosome Profiling analysis showed increased reads densities near the mRNA start site rather than along the mRNA as typical for elongation inhibitors (Nakahigashi et al., 2016). Recent reports and previous observations indicated that tetracyclines could inhibit translation initiation, yet the mechanism remained unknown. Despite the large consensus indicating the Tetracyclines exert their inhibitory function by interfering with translation elongation and some hints on translation initiation, to our knowledge, this is the first detailed report on the mechanism of tetracycline-mediated translation inhibition at a stage other than elongation.

**FIGURE 6:**
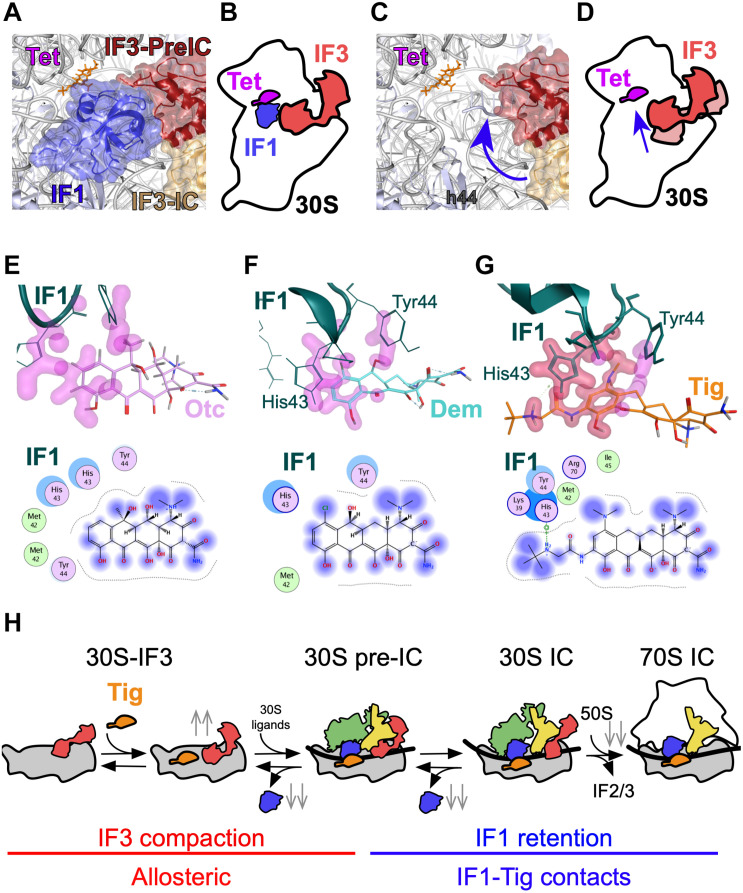
Model of Tetracycline-mediated translation initiation inhibition. **(A)** Structure of the 30S subunit bound to IF1, tetracyclines, and IF3 in two states, 30S–IFs complex (red, PDB: 5LMV), or in the 30S IC (golden, PDB: 5LMN). **(B)** Model of IF3 layout on the 30S–IF1–IF3–Tet complex. **(C)** Structure highlighting IF3 compaction upon binding of Tetracyclines (blue arrow). **(D)** Schematics representing IF3 compaction as a function of Tetracyclines binding. Structural closeup and interaction maps of the **(E)** Otc (pink), **(F)** Dem (aquamarine), and **(G)** Tig (orange) binding pockets in the 30S IC. Residues of IF1 that are in close proximity with Tetracyclines are indicated. Interaction maps for panels **(E–G)**: Blue smudges that are drawn behind tetracycline structures denote the extent of solvent exposure. Arene system contacts involving π–π, π–H, and π–cation interaction are shown in green rings. The dotted outline that surrounds the tetracyclines denotes the distance to the interior pocket. **(H)** Schematics summarizing the mechanism of Tig-mediated translation initiation inhibition through two major effects, IF3 compaction in early complexes (red) and IF1 retention in late complexes (blue). Gray double arrows indicate the increase (up) or decrease (down) of elemental reactions measured here.

We observe that all three tetracyclines tested here induce a conformational change in the 30S-bound IF3, promoting a more compact state of the factor if compared to the absence of the antibiotic. A similar compaction on IF3 was observed upon binding of IF1 (Elvekrog and Gonzalez, 2013; Chulluncuy et al., 2016; Supplementary Figure 6). Indeed, when the binding of Tetracyclines was measured in the presence of IF1, lower compaction, as seen from the amplitudes of the signal, was observed (Supplementary Figure 2). This can be interpreted as the antibiotics promote a similar movement on IF3, at least concerning the directionality and interdomain distance of the factor. IF1 and IF3 have been shown to cooperatively enhance 30S IC formation. In early complex, each initiation factor increases the affinity of the other for the 30S subunit (Milon et al., 2012; Takahashi et al., 2013). In later translation initiation events, both factors are required for kinetically checking the progression toward translation elongation (Milon et al., 2008). The reciprocal enhancement between IF1 and IF3 may be allosterically mediated by the 30S (Gualerzi and Pon, 2015) or by direct contacts between the factors. CryoEM reconstructions showed that factors can contact each other in early complexes (i.e., 30S–IFs), providing a physical explanation of their cooperativity (Hussain et al., 2016). In late complexes, the factors lose their contact points, suggesting that the reciprocal enhancement of functions is more likely mediated by the 30S ribosome (Julián et al., 2011). The conformational change that tetracyclines induce in IF3 is likely to support a rather allosteric molecular network between IF1 in the A site and IF3 in the P site (Figures 6A-D).

The IF3 response to tetracyclines allowed us to calculate the binding kinetics and affinities of the compounds for the 30S with and without IF1 (Figure 2). Our results show cooperativity between IF1 and all three tetracyclines, with an increasing affinity of Tig and Dem for the 30S–IF3–IF1 complex. IF1 reduced the dissociation rate constant *k^–1^* of Tig and Dem (Table 1). The structural analysis showed increased interactions between the 30S and Tig or Dem when IF1 was bound, in addition to direct contacts between the factor and Tig (Figures 6E–G). Thus, the cooperative stabilization and reduction of the drug off rate may be mediated by cooperatively reshaping the A site. Altogether, our data show that the mechanism of Tetracyclines binding does not compete with IF1, albeit both bind the A site. This mechanism differs from that shown for A site binding tRNAs during elongation, where tetracyclines compete with tRNAs for the same site.

From the IF1 perspective, factor dissociation rates from 30S complexes are also compromised, slowed by Tetracyclines (Figure 3). A simplistic explanation entails that the Tetracycline-dependent IF1 stabilization may be driven by direct interactions between the drugs and the factor (Figure 5). Our structural analysis seems to partially support this hypothesis. For instance, the gained contacts of Dem with IF1 during 30S IC compared to the pre-IC could explain the slower dissociation rates. A similar concordance is found for OTC that appears to increase its dissociation rate and lose contacts in the 30S IC. On the other hand, Tig appears to establish contacts with His43 of IF1 in the 30S IC, yet the overall dissociation from this complex is higher than for the 30S pre-IC, lacking the contacts. However, the number of potential contacts between residues in IF1 and Tig in the 30S IC is greater than for Dem or OTC (Figures 6E–G). A solid model where direct contacts explain the kinetic differences likely requires further structural studies, either by crystallography or cryoEM. Alternatively, Tetracyclines may perturb the intramolecular conformational coupling between IF1 and IF3 binding sites. Consistent with this model, A site binders Streptomycin (Str) and Kanamycin (Kan) were also shown to compromise IF3, yet oppositely (Chulluncuy et al., 2016). Str induced an extended layout of IF3 independently of the presence of IF1 in early complexes, whereas the antibiotic induced premature 50S joining to late complexes, largely controlled by IF3 (Milon et al., 2008; Chulluncuy et al., 2016). Thus, rRNA residues in the A site can propagate conformational changes across the 30S to the IF3 binding site, and Tetracyclines, Streptomycin, and Kanamycin may exploit such communication lines (Figure 6). Dem appears to slightly increase the rate of 50S association to 30S ICs, whereas OTC and Tig slow it. Consistently, IF1 dissociation from the corresponding 70S pre-ICs is faster for Dem if compared to the absence of the tetracycline. In contrast, in the presence of OTC, the factor dissociates at slower rates. In the case of Tig, IF1 is retained in the 70S IC, likely contributing to impair aminoacyl-tRNA binding (Figure 6H).

Altogether, our data consistently indicates that all three tetracyclines induce conformational changes in IF3, and IF1 cooperatively enhances their interaction with the 30S (Figure 6H). However, the extent of the effects and the kinetic stability of IF1 are compound- and complex-dependent. Direct contacts between IF1 and Tetracyclines, together with reshaping the A site, likely propagate conformational changes across the ribosome, perturbing important physiological intermediates and finally slowing 70S IC to enter elongation. Whether translation inhibition at the initiation phase predominates over elongation remains an open question. However, the fact that tetracycline increased the number of ribosomes stalled at start sites argues for a predominant inhibition at the initiation phase *in vivo* (Nakahigashi et al., 2016).

## Materials and Methods

### Antibiotics

Oxytetracycline, Demeclocycline, and Tigecycline were purchased from Abcam (Cambridge, MA, United States). Oxytetracycline and Demeclocycline were dissolved in nuclease-free water and Tigecycline in dimethyl sulfoxide to reach a final concentration of 10 mM. They were stored at −20°C for up to 1 month.

### IF1, IF2, and IF3 Expression and Purification

Competent BL21 *E. coli* cells (Mix & Go, Zymo Research, Irvine, CA, United States) were transformed using an expression vector: pET24c for *Inf*A (for IF1), *Inf*B (for IF2), *Inf*C (for IF3 *wild type*), or *Inf*C E166C (for mutated IF3 with a cysteine in the 166 position). Cloned pET24c vectors were purchased commercially (GenScript, Piscataway, NJ, United States). Luria–Bertani broth was used to grow BL21 *E. coli* strains at 37°C. Once the culture reached an OD_600 nm_ of 0.5 U, the protein expression was induced by adding 1 mM of isopropyl β-D-1-thiogalactopyranoside (Thermo Fisher Scientific, Hvidovre, Denmark). The protein expression lasted 3 h, and cells were collected by centrifugation at 5,000 *g* for 10 min at 4°C. Cell pellets were resuspended in lysis buffer (50 mM Hepes pH 7, 100 mM NH_4_Cl, 10 mM MgCl_2_, 10% glycerol, 6 mM β-mercaptoethanol) with protease inhibitors (Merck, Darmstadt, Germany). Then, the cells were lysed by sonication (Fisher Scientific) for 20 cycles (10 s on, 30 s off) at 30% of intensity. Cell lysates were clarified by two rounds of centrifugation at 11,000 *g* for 30 min to remove the remaining cell debris.

For protein purification, cation exchange chromatography was applied for IF1, IF2, and IF3. IF1 and IF3 cell lysates were loaded onto a HiTrap SP HP column (GE Healthcare Life Sciences, Uppsala, Sweden) followed by separation using a 50 mM to 1 M NH_4_Cl gradient. IF3 eluted at 600 mM NH_4_Cl (in buffer 50 mM Hepes pH 7, 10 mM MgCl_2_, 10% glycerol, 6 mM β-mercaptoethanol) with purity above 99% as judged by SDS-PAGE using 15% acrylamide. IF1 eluted at 300 mM NH_4_Cl and required a subsequent purification step to the presence of high molecular weight contaminants. Hence, a filtering step was carried out using an Amicon Ultra 30 kDa centrifugal filter (Merck, Darmstadt, Germany) for 10 min at 14,000 *g* at 4°C. On the other hand, IF2 purification on the cation exchange column was preceded by affinity and anion exchange chromatography (HisTrap HP; GE Healthcare Life Sciences, Uppsala, Sweden). The clarified cell lysate was loaded manually onto the column and washed with wash buffer (20 mM sodium phosphate pH 7.4, 500 mM NaCl, 300 mM imidazole) and eluted with elution buffer (20 mM sodium phosphate pH 7.4, 500 mM imidazole, 100 mM NaCl). Subsequently, IF2 containing fractions were further purified in a Capto Q column using a gradient from 50 mM to 1.5 M NaCl in buffer A (25 mM Tris pH 8, 6 mM 2-Mercaptoethanol, and 5% glycerol). IF2-containing fractions were then concentrated in Amicon centrifugation tubes (Merck, Darmstadt, Germany). IF2 was further purified on a Capto SP HiTrap column (GE Healthcare Life Sciences, Uppsala, Sweden) with buffer F (25 mm Hepes pH 7.1, 6 mM 2-Mercaptoethanol, 5% glycerol) using a 100 mM to 1 M KCl gradient. All three initiation factors were dialyzed in Storage Buffer (25 mM Tris–HCl pH 7.1, 200 mM NH_4_Cl, 10% glycerol, 6 mM 2-Mercaptoethanol) prior to aliquoting and storage at −80°C. Purity was assessed by SDS-PAGE using appropriate acrylamide concentrations and blue Coomassie staining. Bradford protein assay (Biorad, Hercules, CA) was used to measure the concentration of each purified initiation factor.

### Ribosomal Subunits, tRNAs, mRNAs, and Fluorescently Labeled IFs

Purification of bacterial 30S ribosomal subunits was made by sucrose gradients and zonal centrifugation as detailed in Milon et al. (2007). fMet-tRNA^fMet^ was aminoacylated, formylated, and purified by HPLC as described in Milon et al. (2007). Model mRNAs with AUG start codon were chemically and commercially obtained (TriLink Biotechnologies, San Diego, CA, United States) followingg the sequence: AAA CAA UUG GAA UAA GGU aug UUU GGC AAA CGA G. fMet-tRNA^fMet^ (Flu) was produced as detailed in Milon et al. (2007) and kindly provided by Dr. Andrey Konevega. IF3E166C was purified as detailed above for *wt* IF3 and labeled as detailed in Chulluncuy et al. (2016). IF1D4C was purified as for *wt* IF1 and labeled in labeling (25 mM Tris pH 7.1, 100 mM NaCl, 10% Glycerol) using a 20-molar excess of Atto-540Q maleimide (Atto-Tec GmbH, Siegen, Germany) for 45 min at room temperature in motion. The reaction was stopped by adding 6 mM 2-Mercaptoethanol. Labeled IF1 was purified from the dye excess using a HiTrap SP HP column as described above for the purification of the factor.

### Kinetic Experiments and Analysis

All reactions were performed in TAKM_7_ buffer [25 mM Tris–HCl (pH 7.4), 70 mM NH_4_Ac_2_, 30 mM KCl, 7 mM MgCl_2_], and 30S subunits were activated inTAKM_21_ (TAKM_7_ buffer with 1/10th volume 140 mM MgCl_2_) at 37°C for 30 min. Then, each reaction solution was centrifuged at 15,000 × *g* for 10 min at 20°C and loaded to the stopped-flow instrument. Fluorescent measurements were performed using an SX20 stopped-flow apparatus (Applied Photophysics, Surrey, United Kingdom). Each pair of reactants was mixed rapidly in equal volumes (90 μl). To excite the donor fluorophore (Alexa-488 or Fluorescein), a monochromatic LED (470 nm) (Applied Photophysics, Surrey, United Kingdom) was used. Alexa-488 was used as a donor fluorophore for the IF3_DL_, IF3_488_, and fMet-tRNA (Flu) FRET signals. An optical cut-off filter 515 nm was used preceding the Photo Multiplier to measure donor emission fluorescence. Typical stopped-flow experiments used the following concentrations if the component was present: 0.05 μM 30S subunits, 0.15 μM IFs, 0.15 μM fMet-tRNA^fMet^, 0.3 μM mRNA, 0.2 mM GTP in TAKM_7_. The FRET donor component was always kept in a one-to-one ratio with 30S subunits. All experiments used 100 μM of the indicated tetracycline unless otherwise stated. IF1_540Q_ chase experiments used a 10-fold excess of unlabeled IF1 in the stopped-flow apparatus. Light scattering experiments used monochromatic light at 430 nm wavelength, and the scattered light was measured at an angle of 90° without a filter. Fluorescence and light scattering data were collected using 1,000 points in logarithmic mode. For each reaction, five to eight replicates were recorded and averaged. The resulting time traces were analyzed using Prism 8.0 (GraphPad Software, La Jolla, CA, United States) program with the appropriate equations. Non-linear regression equations (Eqs 1, 2) were used accordingly.

(1) $$\text{F}={\text{F}}_{0}+{\text{F}}_{1}*\text{exp}\left(-k_{\mathrm{app1}}*\text{t}\right)$$

(2) $$\text{F}={\text{F}}_{0}+{\text{F}}_{1}*\text{exp}\left(-k_{\mathrm{app1}}*\text{t}\right)+{\text{F}}_{2}*\text{exp}\left(-k_{\mathrm{app2}}*\text{t}\right)$$

The microscopic constants *k*_1_, *k*_–1_, *k*_2_, and *k*_–2_ for Tig binding to 30S–IF3_DL_ complexes were calculated by plotting both the sum and product of the apparent rates *k*_app1_ and *k*_app2_ for each titration and analyzing the resulting linear relationship. Briefly, taking *A* as the linear regression of the sum of *k*_app1_ and *k*_app2_, and *B* as the linear regression of the product of *k*_app1_ and *k*_app2_, kinetic parameters were determined as follows *k*_1_ = slope(A); *k*_–1_ = intercept(A) − (slope(B)/*k*_1_); *k*_–2_ = intercept(B)/*k*_–1_; *k*_2_ = intercept(A) − *k*_–1_ − *k_–2_.* Dissociation constants (*K*_D_) were calculated using the following equation: *K_D_* = (*k*_–1_ × *k*_–2_ × *k*_1_^–1^)/(*k*_–2_ + *k*_2_).

### *In silico* Modeling

Two procedures were performed in the molecular modeling section: Structure-based drug design of tetracycline derivatives and the building of initiation and pre-initiation complexes. Tetracycline derivatives were modeled on the ribosome from *Thermus thermophilus* using the structure-based drug design tool implemented in the MOE program. The approach allows users to explore protein–ligand interactions and manually constructing novel compounds in protein binding sites. Novel compounds are energy minimized inside the binding site and ranked by binding free energy and affinity calculations. The 30S ribosome structure bound to Tigecycline was obtained from PDB crystal ID: 4YHH (X-ray diffraction with 3.4 Å) (Schedlbauer et al., 2015). The crystal structure 4YHH was prepared using the Quick Prep tool, which involves important steps such as reparation of structural problems, the rebuild of the hydrogen bond network, three-dimensional protonation (3D), and energy minimization. Then, the prepared crystal 4YHH containing Tigecycline as a starting point was edited in order to obtain demeclocycline and oxytetracycline molecules. After that, energy minimizations were performed to refine the structure and avoid steric clashes. The models obtained were analyzed manually using the LigPlot module, which generates representations of the molecule and its 2D ligand as well as its interactions with the option Ligand Interaction. To ensure the reproducibility of the molecular modeling protocol (internal control), the same procedure was performed on a similar crystal obtained using the same method with 4.5 Å resolution, which contained 30S and Tetracycline (PDB ID: 1I97) (Pioletti et al., 2001). In order to analyze the molecular interactions of tetracyclines in the formation of initiation complexes, previous models of the 30S and Tetracyclines were aligned to the pre-initiation and initiation complex using the PyMOL program (PDB ID: 5LMQ and PDB ID: 5LMV, respectively) (Hussain et al., 2016). Therefore, each tetracycline derivative modeled was produced from two crystals: Tetracyclines from PDB ID: 4YHH and initiation complex crystal PDBs: 5LMQ, 5LMV.

## Data Availability Statement

The original contributions presented in the study are included in the article/Supplementary Material, further inquiries can be directed to the corresponding author/s.

## Author Contributions

MV-R and VB performed measurements. PM conceived the project. All authors analyzed the data, actively participated in writing the manuscript, and contributed to the elaboration of figures.

## Conflict of Interest

The authors declare that the research was conducted in the absence of any commercial or financial relationships that could be construed as a potential conflict of interest.
