# Supplementary material for: A Complementary Mechanism of Bacterial mRNA Translation Inhibition by Tetracyclines
Source: Front Microbiol. 2021 Jun 28;12:682682. doi: 10.3389/fmicb.2021.682682 (PMC8273347; doi:10.3389/fmicb.2021.682682)
Supplement: Supplementary file 1 [file Data_Sheet_1.PDF]

Supplementary Material for:

A complementary mechanism of bacterial mRNA translation inhibition by Tetracyclines

Victor Barrenechea[1,2], Maryhory Vargas-Reyes[1], Miguel Quiliano[1], and Pohl Milón[1,*]

[1] Centre for Research and Innovation, Faculty of Health Sciences, Universidad Peruana de Ciencias Aplicadas (UPC), Lima 15023, Peru

[2] Postgraduate Unit, Medicine Faculty, Universidad Nacional Mayor de San Marcos, Lima 15001, Peru

*** Correspondence:**
Pohl Milón
pmilon@upc.edu.pe

Supplementary Figures

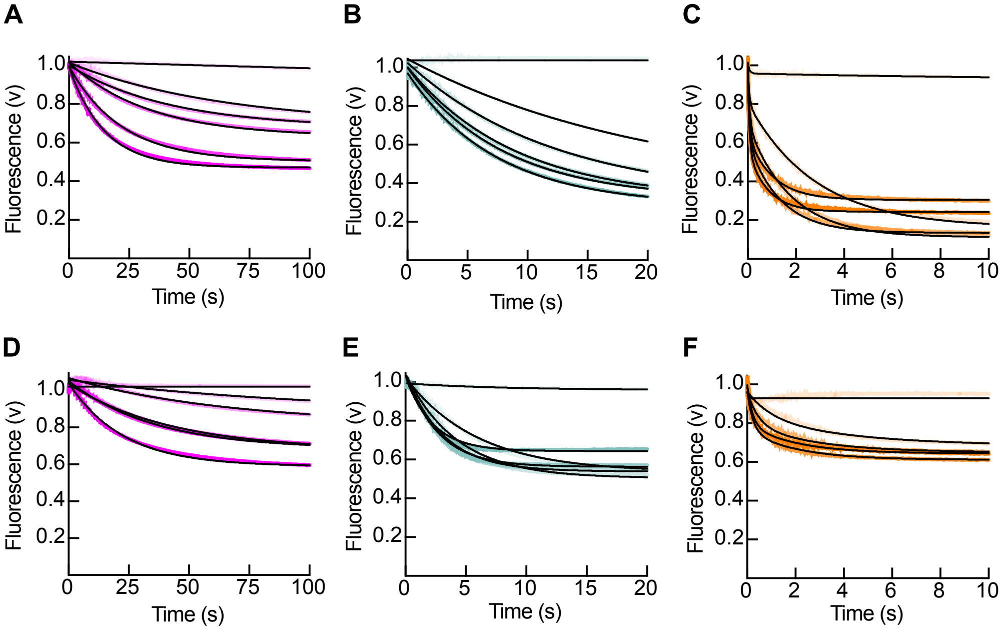

Supplementary Figure 1. Tetracyclines binging to 30S-IF3$_{DL}$ and 30S-IF1-IF3$_{DL}$ complexes (Related to Figure 2). Time courses of Otc **(A)**, Dem **(B)**, and Tig **(C)** biding to 30S-IF3$_{DL}$ complexes at increasing concentrations of the increasing concentrations of the respective antibiotic. The intensity of colors relate to antibiotic concentration (5 – 100 µM for Otc, 100 – 500 µM for Dem or Tig). **(D-F)** Timecourses of Tetracyclines binding to 30S-IF1-IF3$_{DL}$. Each time course represents the average of 5-8 replicates. Continuous black lines show the fitting with appropriate exponential equations.

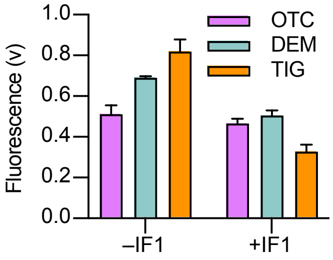

Supplementary Figure 2. Comparison of the maximal FRET change of IF3DL in 30S complexes with or without IF1 as function of Otc (pink), Dem (aquamarine), and Tig (orange).

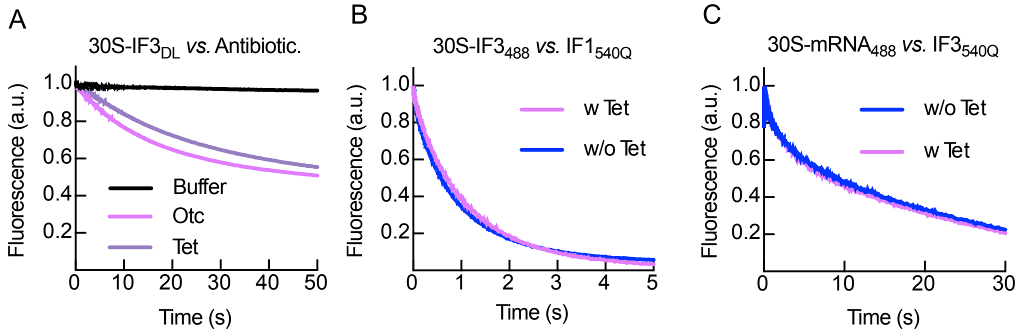

Supplementary Figure 3. Binding of Otc and Tet result in similar IF3DL conformational changes and Tetracyclines do not perturb IF1, nor IF3 binding.

(A) Time traces comparing the binding of 100 µM Tet or Otc to 30S-IF3DL complexes. **(B)** Time traces of $IF1_{540Q}$ binding to $30S\text{-}IF3_{488}$ complexes in the presence (pink) of absence (blue) of Otc. **(C)** Time traces of $IF3_{540Q}$ binding to $30S\text{-}mRNA_{488}$ complexes in the presence (pink) or absence (blue) of Otc. Each time course represents the normalized average of 5-8 replicates.

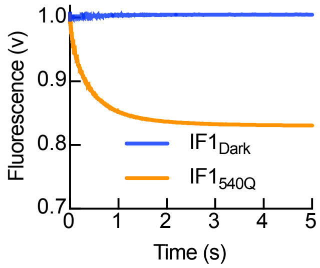

Supplementary Figure 4. Controls for the FRET between $IF3_{488}$ and $IF1_{540Q}$.

Binding of 0.15 µM unlabeled IF1 (blue) or $IF1_{540Q}$ (orange) to 0.05 µM $30S\text{-}IF3_{488}$ complexes was measured in the stopped-flow apparatus. The reduction of fluorescence in time corresponds to the proximity of the quencher in $IF1_{540Q}$ near the donor dye in $IF3_{488}$ rather than a fluorescence change in the donor surroundings due to IF1. Each time course represents the normalized average of 5-8 replicates.

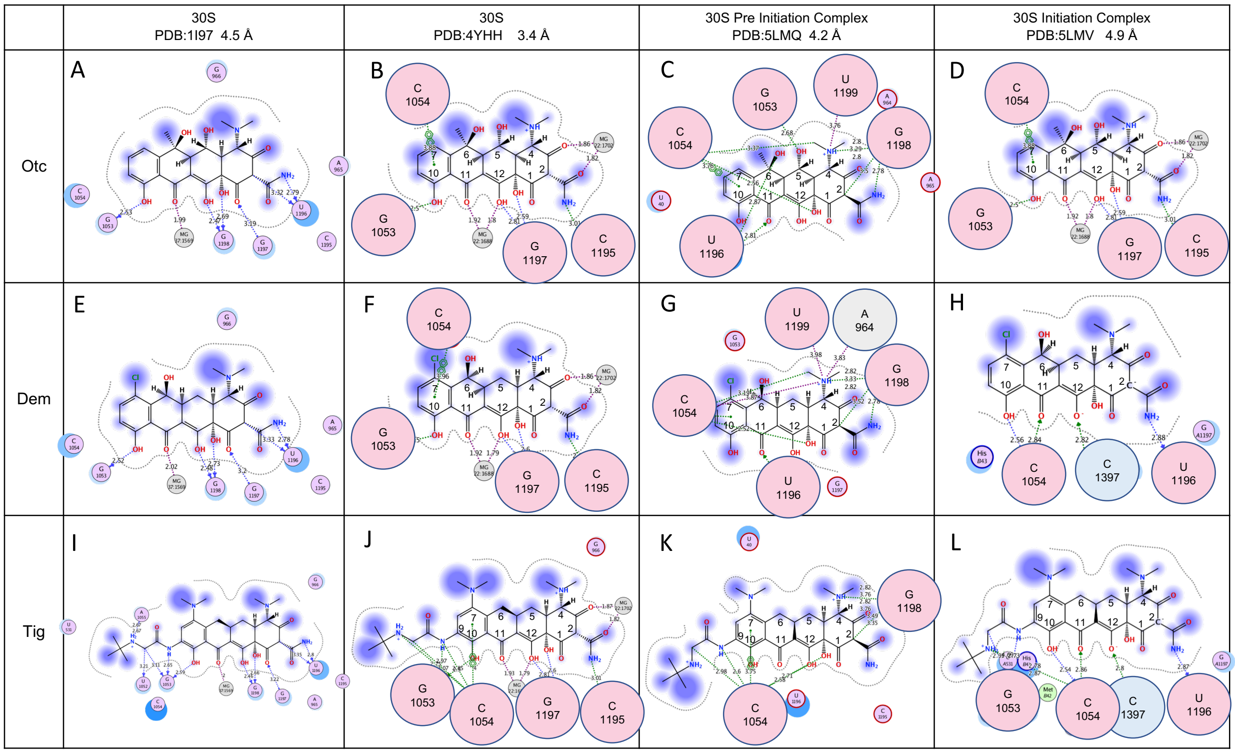

Supplementary Figure 5. Interaction map between Tetracyclines and 30S initiation complexes. **(A-D)** Oxytetracycline, **(E-H)** Demeclocycline, and **(I-L)** Tigecycline. Blue smudges that are drawn behind tetracycline structures denote the extent of solvent exposure. The green arrow denotes donor or acceptor sidechain, blue arrows denote donor or acceptor backbone. Arene system contacts involving π- π, π-H and π-cation interaction are shown in green rings. The dotted outline that surrounds the tetracyclines denote the distance to the interior pocket

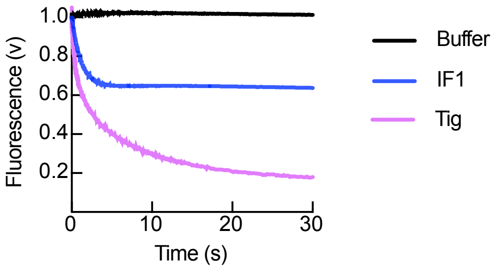

Supplementary Figure 6. IF1 and Tig promote similar rearrangements in the 30S-bound $IF3_{DL}$

Time courses of 0.15 µM IF1 (blue) or 100 µM Tig (pink) binding to 0.05 µM 30S-$IF3_{DL}$ complexes. A dilution control (black) consisted in mixing 30S-IF3 complexes with buffer in the stopped-flow apparatus. Each time course represents the normalized average of 5-8 replicates.
